# Assessing the Utilization of Postnatal Services Among Mothers: A Cross-Sectional Study

**DOI:** 10.7759/cureus.47000

**Published:** 2023-10-13

**Authors:** Sunvir Kaur Rai, Simmi Oberoi, Rajinder Singh Balgir, Dharminder Ahir, Harpreet Singh

**Affiliations:** 1 Community Medicine, Government Medical College, Patiala, IND; 2 Health and Family Medicine, Community Health Center, Patiala, IND

**Keywords:** cross-sectional study, utilization, postnatal care, health care utilization, health services accessibility

## Abstract

Background: Women in the postnatal period are a special group with a high risk to health. Providing good quality postnatal care can help reduce maternal morbidity and mortality and improve the quality of life. The objective of the study was to assess the utilization of the postnatal services provided to mothers and to find the factors affecting the utilization of these services.

Materials and methods: A cross-sectional study where 154 mothers from Patiala were interviewed regarding postnatal services using a pretested semi-structured questionnaire.

Results: A total of 92.9% of mothers (95%CI=88.76-97.04) availed postnatal care with a multi-purpose health worker-female (MPHW-F) and an accredited social health activist (ASHA (U)) as the main providers. Only 47.4% of mothers (95%CI=39.35-55.45) had visited a doctor for a postnatal check-up. Mother’s education, type of family, place, and type of delivery were significantly associated with the number of visits to the doctor during the postnatal period. Thirty-nine (25.3%) mothers (95%CI=18.3-32.3) reported a health problem in the period, out of which only 32 mothers had taken treatment for their health problems. Mothers who were visited by MPHW-F in the postnatal period had fewer morbidities as compared to those who were not visited by MPHW-F *(χ*^2^=7.697; df=2; p value=0.02).

Conclusions: Working women with cesarean section delivery in the private sector reported more utilization of postnatal services. These women had higher education levels and belonged to joint families. More visits by MPHW-F were associated with fewer health problems. A multi-pronged approach, targeting individuals, families, and communities, may be necessary to improve postnatal care service utilization rates.

## Introduction

The experience of becoming a mother is one of the most profound transformations in women’s lives. Often, while bearing and looking after the children and family, the mother’s health is compromised. This can lead to impairments, disabilities, and even deaths in extreme situations. Special care must be provided to mothers, especially new mothers, to promote their long-term physiological and emotional well-being [1].

Women in the postnatal period form a special risk group who are most vulnerable during the six-week interval period following the delivery of a child [1]. Certain complications may affect the mother’s health during the postpartum period, which should be recognized early and dealt with promptly. These include postpartum hemorrhage, puerperal sepsis, breast complications, urinary tract infections, thrombophlebitis, and psychological problems [2].

India accounts for 7.5% (24,000) of all global maternal deaths [3]. The Sample Registration System (SRS) (2019-21) reports that the maternal mortality rate in India is 97 per lakh live births [4]. Hemorrhage (47%) and sepsis (12%) are India's major causes of maternal mortality [5].

Medical supervision of mothers within the first 24 hours after delivery can help in identifying and addressing the problems that may occur [6]. After discharge from the hospital, outreach services are provided to the mother through a multi-purpose health worker-female (MPHW-F) and an accredited social health activist (ASHA (U)) [7]. Mothers are also advised on breastfeeding, nutrition, family planning, and hygiene [2].

These measures help reduce maternal morbidity and mortality, improve the quality of care, enhance the satisfaction of beneficiaries, and provide respectful maternity care to all pregnant women attending public health facilities [8].

Despite being one of the first countries to launch maternal health programs, India is still struggling with high maternal mortality and morbidity compounded by low utilization of services. A National Family Health Survey 5 (2019-21) reported that in India 79% of mothers received postnatal care from a doctor/nurse/lady health visiter/auxiliary nurse midwife/midwife/other health personnel within two days of delivery [9]. This figure for Punjab state is 84.7%, and there is scope for more improvement in outside states [10].

Utilization of postnatal care has been related to knowledge about its importance, level of education, access to health services, counseling of postnatal care in institutional deliveries, and women’s tendency to give priority to the health needs of their infants over their own [11]. The present study was, thus, planned to know the utilization of the postnatal services provided to mothers and to find the factors affecting the utilization of services.

## Materials and methods

Study design

A community-based observational cross-sectional study was conducted in the urban field practice area of the Department of Community Medicine, Government Medical College, Patiala, Punjab, India, from January 1, 2017, to December 31, 2017.

Study participants

The study included all the mothers who had completed 28 weeks of gestation and delivered either a live-born or a stillborn child. Mothers who were registered in the field practice area but had not delivered and stayed in the study area during the postnatal period were excluded.

Sample size

**Figure 1:**
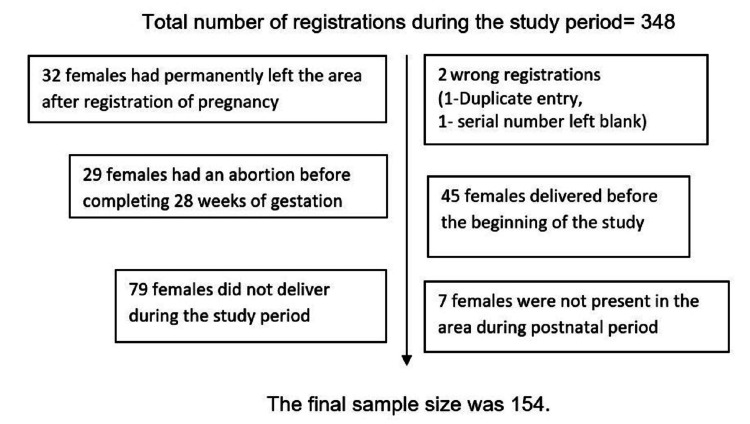
Sample size calculation

The list of mothers who had completed their six weeks of the postnatal period was prepared from the record of the MPHW-F. Purposive sampling was applied, and the final sample size was finalized at 154. The calculation is presented in Figure 1.

Data collection

Ethical clearance was obtained before data collection. An urban training health center was visited twice a week for the purpose of data collection. The mothers were contacted at their residences for data collection. Locked houses during the first visit were revisited, and interviews were conducted.

Data for the study were collected on a pretested, validated, and semi-structured questionnaire through face-to-face interviews after obtaining written informed consent.

The questionnaire consisted of the socio-demographic profile of the mother and details of the postnatal care utilized by her after the present delivery. Factors affecting the utilization of services were recorded. The roles of MPHW-F and ASHA (U) in the provision of postnatal care were assessed. The mother’s discharge summary and treatment charts were reviewed for reporting health problems. Components related to the mother’s health only were taken into consideration. Postnatal services bearing on the infant’s health were not considered due to limited resources (time and finance).

Data entry and analysis

All the collected data were compiled and analyzed in Microsoft Excel Sheet 2016 (Microsoft Corporation, Redmond, Washington). Descriptive analysis in the form of numbers and percentages was calculated and reported for categorical variables. Mean with standard deviation was calculated for continuous variables. The chi-square test was used to determine the association between qualitative variables. The values were significantly associated if the P value was less than 0.05.

## Results

A total of 154 mothers in the field practice area were interviewed, and their socio-demographic and obstetric details were recorded (Table 1). The mean age of postnatal mothers was 26.656±4.05 years, and the range was 21-38 years. Additionally, 64.3% of mothers belonged to joint families, only 24.7% were working among them, while the rest were housewives.

**Table 1:** Profile of postnatal mothers (n=154)

Variables	No. of Subjects	Percentage
Place of residence		
Guru Nanak Nagar*	76	49.3%
Kashmiri Gurudwara	38	24.7%
MCH Tripuri	34	22.1%
Gurubhawan	6	3.9%
Age (in years)		
Up to 25	60	39.0%
25-30	67	43.5%
30 and above	27	17.5%
Educational status		
Illiterate	39	25.3%
Up to Senior Secondary	62	40.3%
Graduate & above	53	34.4%
Duration of stay in health facility after delivery		
Up to 12 hours	26	16.9%
12-24 hours	15	9.7%
24-48 hours	18	11.7%
48 hours and more	95	61.7%

Moreover, 57.1% of mothers had delivered in government facilities. There were no home deliveries in the area during the period of data collection. Most of the mothers (64.3%) had delivered normally per vaginum.

While 92.9% of mothers (95%CI=88.76-97.04) had availed postnatal care during six weeks following delivery, 11 mothers did not receive any home visits for postnatal care. The most common perceived barrier to utilization of postnatal care services was being not aware of its need (n= six), followed by no health problem occurring in puerperium (n=three) and “no one to take care of children at home in their absence” (n= two).

The main providers of postnatal care were MPHW-F and ASHA (U) in 35 (22.7%) mothers (95%CI=15.94-29.46), followed by a doctor and an MPHW-F in 32 (20.8%) mothers (95%CI=14.26-27.34), only an MPW-F in 28 (18.2%) mothers (95%CI=11.98-24.42), only a doctor in 19 (12.3%) mothers (95%CI=7.00-17.59), a doctor along with an MPHW-F and ASHA (U) in 18 (11.3%) mothers (95%CI=6.19-16.4), only an ASHA (U) in seven (4.5%) mothers (95%CI=1.16-7.84), and a doctor along with an ASHA (U) in four (2.6%) mothers (95%CI=0.04-5.16).

The study shows that the majority 52.6% of mothers (95%CI=44.56-60.65) did not receive any postnatal check-ups from a doctor, 36.36% of mothers (95%CI=28.61-44.11) had one to two postnatal visits, and only 11.04% mothers (95%CI=5.91-16.17) had ≥3 visits. Factors affecting the number of visits to a doctor in the postnatal period are presented in Table 2.

**Table 2:** Distribution of variables (mother’s) vis a vis number of visits X2= chi-square test; S = significant; NS= not significant

Number of Visits to a Doctor in Postnatal Period	0	1-2	≥ 3	Total	P values
Variables	Number (%age)	Number (%age)	Number (%age)	Number (%age)	
Type of Family: Nuclear Family	36 (65.5%)	13 (23.6%)	6 (10.9%)	55 (100.0%)	χ^2^=6.0513, p= 0.039 (S)
Joint Family	45 (45.5%)	43 (43.4%)	11 (11.1%)	99 (100.0%)	
Education Status: Illiterate	26 (66.6%)	11 (28.2%)	2 (5.2%)	39 (100.0%)	χ2=20, p= <0.01 (S)
Up to Senior Secondary	39 (62.9%)	20 (32.3%)	3 (4.8%)	62 (100.0%)	
Graduate & Above	16 (30.2%)	25 (47.2%)	12 (22.6%)	53 (100.0%)	
Occupation: Housewife	66 (56.9%)	40 (34.5%)	10 (8.6%)	116 (100.0%)	χ2=4.600, p=0.1 (NS)
Working	15 (39.5%)	16 (42.1%)	7 (18.4%)	38 (100.0%)	
Place of Delivery: Government Facility	59 (67.1%)	25 (28.4%)	4 (4.5%)	88 (100.0%)	χ2=19.565, p≤ 0.01(S)
Private Facility	22 (33.3%)	31 (46.9%)	13 (19.8%)	66 (100.0%)	
Type of Delivery: Normal Vaginal Delivery	69 (69.7%)	22 (22.2%)	8 (8.1%)	99 (100.0%)	χ2=32.85, p≤ 0.01(S)
Cesarean Section	12 (21.8%)	34 (61.8%)	9 (16.4%)	55 (100.0%)	

**Figure 2:**
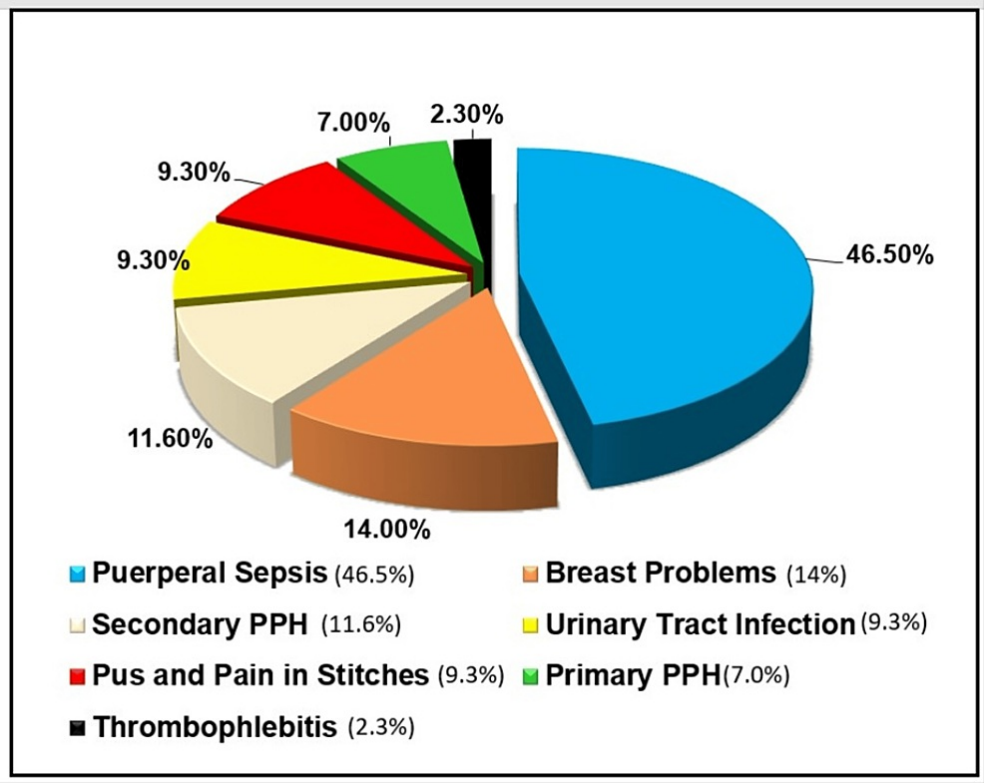
Distribution of maternal morbidities during postnatal period (n= 43)

The study reported that 39 (25.3%) mothers (95%CI=18.3-32.3) had health problems in the postnatal period. Four mothers had more than one health problems. Thus, 43 health problems were detected in 39 mothers. No maternal deaths were reported during the study period. The distribution of maternal morbidities during the postnatal period is presented in Figure 2.

Distribution of health problems vis a vis MPHW-F and ASHA (U) home visits during the postnatal period are presented in Table 3.

**Table 3:** Distribution of health problems vis a vis MPHW-F and ASHA (U) home visits during the postnatal period X2= chi-square test; S = significant; NS= Not significant ASHA (U) covered only one area in the study (n=76)

Number of visits by MPHWF	0	1-2	≥ 3	Total	P value
Health problems in the postnatal period	Number (%)	Number (%)	Number (%)	Number (%)	
Yes	17 (43.59%)	5 (12.82%)	17 (43.59%)	39 (100%)	X^2 ^= 7.69 p = 0.02 (S)
No	24 (20.87%)	21 (18.26%)	70 (60.87%)	115 (100%)	
Total	41 (26.62%)	26 (16.88%)	87 (56.49%)	154 (100%)	
Number of visits by ASHA	0	1-2	≥ 3	Total	P value
Health problems in the postnatal period	Number (%)	Number (%)	Number (%)	Number (%)	
Yes	1 (5.26%)	1 (5.26%)	17 (89.48%)	19 (100%)	X^2 ^= 2.3 p= 0.316 (NS)
No	11 (19.29%)	4 (7.03%)	42 (73.68%)	57 (100%)	
Total	12 (15.79%)	5 (6.58%)	59 (77.63%)	76 (100%)	

While 32 mothers had taken treatment for health problems in the postnatal period, seven women did not take any treatment. Out of these seven, three mothers believed in traditional care, two mothers had to attend household activities, one mother felt that no treatment was necessary, and one mother did not take any treatment because the health facility was far away.

**Figure 3:**
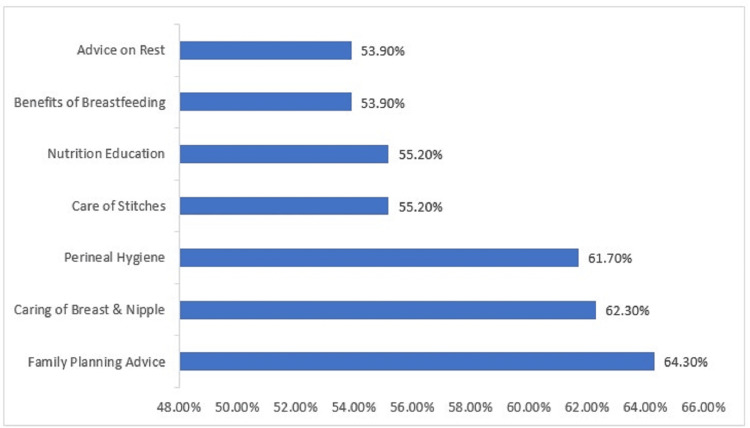
Varying advice received by mothers from healthcare workers in relation to postnatal care

Varying advice received by mothers in relation to postnatal care is given in Figure 3. During the postnatal period, iron and folic acid tablets were consumed daily by 74 (48.0%) mothers, sometimes by 30 (19.5%) mothers, and never by 50 (32.5%) mothers.

## Discussion

Postnatal care is essential in maintaining and promoting the health of the mothers and provides an opportunity for health professionals to identify, monitor, and manage health problems that may develop in the mother during the postnatal period [12].

In the present study, most of the women (92.9%) received postnatal care during the six weeks following delivery. This figure is comparable to NFHS-5 (2019-21) data of Punjab, which revealed that 84.7% of mothers received postnatal care from a doctor/nurse/LHV/ANM/midwife/other health personnel within two days after delivery [9]. A similar result was observed in a study by Tiwari et al. in Gorakhpur where only 7.1% of mothers did not have any postnatal check-ups [13]. This is probably due to increased efforts of the government through the RMNCH+A program. However, in our study, only half of the mothers received the recommended number of four visits from an MPHW-F (56.5%) and seven visits from an ASHA (55.3%). Therefore, there is a need for healthcare providers and policy-makers to develop targeted interventions and make focused efforts to reach beneficiaries for postnatal services.

The study also revealed that, out of 11 mothers who did not utilize postnatal care, most of them (six) were not aware of the postnatal check-ups, followed by three mothers who did not feel the need for postnatal examinations because no health problems occurred in the puerperium, and two mothers did not have anyone else to take care of children at home in their absence. An MPHW-F or ASHA (U) did not visit these mothers at their residences. Lack of knowledge in mothers is the most common reason for non-utilization of postnatal care. This was also concluded in studies by Mahajan (74.05%) [6], Uppadhaya et al. in Rajasthan (90.55%) [14], and Bhattacherjee et al. in West Bengal (94.6%) [15]. The strategic use of social and behavior change communication methods must be employed by providers from the antenatal period to bring about increased awareness regarding the necessity of postnatal care. It must be stressed that postnatal care is a must even if mothers are not experiencing any health problems.

In the present study, mothers received postnatal care from multiple healthcare providers. The main providers of postnatal care were an MPHW-F and ASHA (U) in 22.7% of females, followed by doctors and MPHW-F in 20.8% of females. This is in concurrence with a study by Malik et al. in rural Haryana, which revealed that the majority of the mothers received postnatal care from an ANM/ASHA [16]. On the other hand, a study by Uppadhaya et al. in Jodhpur summarized that doctors were the main providers of maternal services, followed by nurses [14].

The number of subsequent visits to doctors in the postnatal period is significantly associated with the type of family (Table 2). Females from joint families visited doctors more than females from nuclear families. Similar results were reported by Paudel et al. in rural Belgaum [17] and Venkatesh et al. in Karnataka [18]. Studies by Uppadhaya et al. and Sharma et al. did not find any significant association between the type of families with postnatal care utilization [14,19].

The present study revealed that maternal literacy is significantly associated with the number of postnatal visits to doctors (Table 2). The number of doctor visits increased with the increase in mothers' education level. Other studies by Paudel et al., Chimankar et al., and Iyengar also reported similar findings [17,20,21]. The education of mothers is an important factor contributing to postnatal care utilization. Illiteracy and low levels of education may be responsible for a lack of knowledge regarding of the benefits of postnatal care. Prusty et al. opined that the poor utilization among illiterate women may be probably due to ignorance of the benefits of healthcare utilization owing to illiteracy [22].

The present study revealed that, although the level of utilization of health services by working women was more than by housewives, it was not statistically significant (Table 2). However, studies by Uppadhaya et al. and Sharma et al. reported that housewives utilized postnatal care significantly more than working women [14,19].

The number of visits to doctors in the postnatal period was significantly associated with the place of delivery of the mother (Table 2). Mothers from private facilities tend to have more visits to doctors as compared to mothers delivering in government facilities. This might be associated with the package system provided by private facilities and the paying capacity of the beneficiaries.

The number of visits to doctors in the postnatal period is significantly associated with the type of delivery of the mother (Table 2). Mothers who had cesarean section tend to have more visits than mothers having normal vaginal deliveries. This result is in concordance with the study by Mahajan et al. [6]. Females with cesarean section stay at the health facility for a longer duration as compared to those with normal vaginal delivery. Longer duration means more exposure to skilled personnel and hence increased awareness regarding service utilization.

Figure 2 of the present study reveals that the most commonly reported health problem in the postnatal period was puerperal sepsis (20), followed by breast problems (six), secondary PPH (five), urinary tract infection (four), pain and pus in stitches (four), primary PPH (three), and thrombophlebitis (one).

A study by Iyengar reported that life-threatening postpartum morbidities were detected in 7.6% of women, 4.9% of mothers had breast-related conditions, 4.5% of mothers had perineal conditions, 4% of mothers had fever, and 0.6% had UTI [21]. Paudel et al. reported that the common health problems perceived by them were puerperal sepsis with fever (35.1%) and mastitis (10.6%) [17]. A study by Vyas et al. in Udupi reported that urinary and breast problems were present in <10% of the participants [23].

In the present study, seven mothers did not receive any treatment for health problems in the postnatal period. Among these, three believed in traditional care, two mothers had to attend household activities, one mother felt that no treatment was necessary, and one mother did not take any treatment because the health facility was far away. In a study by Paudel et al. in Belgaum in Karnataka, about 46.8% of women with health problems took medicine with the advice of nurses, 16.4% did self-medication without consulting any health worker, while 12.3% did not do anything about the health problem [17].

People's health behavior changes over time through the process of acquiring new information and knowledge about their health and its care, which leads them to form new opinions, attitudes (favorable and unfavorable), and acceptance and rejection in real-life situations [24]. Thus, giving correct information to the clients is of utmost importance. Figure 3 of the present study shows that only one-third of the mothers received advice on family planning, caring for breast and nipple, and perineal hygiene, and about half of the females received advice on the care of stitches, nutrition education, benefits of breastfeeding, and rest.

Lodhiya et al.'s study revealed that 38.07% of health workers advised postnatal mothers about the benefits of breastfeeding, 62.39% on family planning, and 53.21% on maternal nutrition [25]. Paudel et al. found in their study that 89% of mothers got advice on diet, 71.1% on family planning, and 79.7% related to the care of breasts and nipples [17]. Fathima et al.’s study revealed that 83.6% of women received advice on breastfeeding, 58.4% on maternal nutrition, and only 21.2% on contraceptive use [26]. Uppadhaya et al. in their study observed that 96.15% of mothers received advice on diet and 92.3% on family planning (92.31%) [14]. Agarwal et al. found that only 39.1% instructed mothers on the health benefits of breastfeeding [27]. Singh et al. reported that only 65.2% of the health workers discussed family planning methods, and only 43.4% of health workers provided appropriate counseling on diet [28].

In the present study, half of the mothers (48.1%) had consumed iron and folic acid tablets (IFA tablets) daily, whereas 32.5% of mothers had never consumed them. In the study by Paudel et al., 89.0% of mothers consumed IFA tablets during the postnatal period [17].

The present study found that, in spite of 92.9% coverage for postnatal services, there was one female who had serious health problems but was missed for postnatal care. There were six females who had received postnatal care but did not take treatment for their health problems. This shows that even enough postnatal care provision is not enough to achieve our targets. Timely, adequate, and door-to-door provision of healthcare will help in addressing these missed mothers. Repeated training, intense monitoring, and supportive supervision of the services provided by healthcare workers are required to prevent adverse maternal outcomes. Targeting these missed mothers will help us in achieving sustainable development goals.

Limitations

This study reports some potential limitations that must be understood in light of the results. The present study was a cross-sectional study. It relied on maternal recall and may be subject to bias. This study took place in one area of Patiala City, India, so the findings may not be generalizable. Despite these limitations, the study provides important information on the utilization of maternal healthcare services and contributes to identifying barriers to post-partum care.

## Conclusions

The study reveals that, due to the increased availability and accessibility of maternal healthcare services, almost all mothers had a postnatal check-up. However, the number falls to half when it comes to the recommended number of visits by an MPHW-F and ASHA (U). Working mothers of joint families with higher levels of education who delivered in private facilities through cesarean section had higher rates of utilization of postnatal services than their counterparts. More visits by an MPHW-F were associated with fewer health problems. Early detection and timely treatment of health problems should be the focus of the home visits by grassroots-level workers. A multi-pronged approach, targeting individuals, families, and communities, may be necessary to improve postnatal care service utilization rates.
